# Origin, evolution and classification of type-3 copper proteins: lineage-specific gene expansions and losses across the Metazoa

**DOI:** 10.1186/1471-2148-13-96

**Published:** 2013-05-01

**Authors:** Felipe Aguilera, Carmel McDougall, Bernard M Degnan

**Affiliations:** 1 Centre for Marine Science, School of Biological Science, The University of Queensland, Brisbane, Queensland, 4072, Australia

**Keywords:** Type-3 copper proteins, Catechol oxidase, Tyrosinase, Tyrosinase-related protein, Hemocyanin, Gene expansion, Gene loss

## Abstract

**Background:**

Tyrosinases, tyrosinase-related proteins, catechol oxidases and hemocyanins comprise the type-3 copper protein family and are involved in a variety of biological processes, including pigment formation, innate immunity and oxygen transport. Although this family is present in the three domains of life, its origin and early evolution are not well understood. Previous analyses of type-3 copper proteins largely have focussed on specific animal and plant phyla.

**Results:**

Here, we combine genomic, phylogenetic and structural analyses to show that the original type-3 copper protein possessed a signal peptide and may have been secreted (we designate proteins of this type the α subclass). This ancestral type-3 copper protein gene underwent two duplication events, the first prior to the divergence of the unikont eukaryotic lineages and the second before the diversification of animals. The former duplication gave rise to a cytosolic form (β) and the latter to a membrane-bound form (γ). Structural comparisons reveal that the active site of α and γ forms are covered by aliphatic amino acids, and the β form has a highly conserved aromatic residue in this position. The subsequent evolution of this gene family in modern lineages of multicellular eukaryotes is typified by the loss of one or more of these three subclasses and the lineage-specific expansion of one or both of the remaining subclasses.

**Conclusions:**

The diversity of type-3 copper proteins in animals and other eukaryotes is consistent with two ancient gene duplication events leading to α, β and γ subclasses, followed by the differential loss and expansion of one or more of these subclasses in specific kingdoms and phyla. This has led to many lineage-specific type-3 copper protein repertoires and in some cases the independent evolution of functionally-classified tyrosinases and hemocyanins. For example, the oxygen-carrying hemocyanins in arthropods evolved from a β-subclass tyrosinase, whilst hemocyanins in molluscs and urochordates evolved independently from an α-subclass tyrosinase. Minor conformational changes at the active site of α, β and γ forms can produce type-3 copper proteins with capacities to either carry oxygen (hemocyanins), oxidize diphenols (catechol oxidase) or *o*-hydroxylate monophenols (tyrosinases) and appear to underlie some functional convergences.

## Background

Copper is an essential cofactor in a diversity of biological oxidation-reduction reactions due to its existence in either a reduced (Cu^+^) or an oxidized (Cu^2+^) state [1-3]. Oxidized copper preferentially coordinates with oxygen in aspartic and glutamic acids or with the imidazole nitrogen group in histidines [3], allowing interactions with a wide spectrum of proteins. Copper-binding proteins are present in the three domains of life [4-7] and are divided into three classes based on their spectroscopic properties and geometric structure of the active site: type-1 or blue copper proteins, which are involved in electron-transfer (e.g., plastocyanin, azurin and halocyanin); type-2 or non-blue copper proteins, which form part of the oxidoreductase family (e.g., galactose oxidase) [1,2,8]; and the type-3 or binuclear copper protein family, which comprises genes encoding tyrosinase, tyrosinase-related proteins, catechol oxidase and hemocyanin.

Tyrosinases and catechol oxidases, both commonly known as phenoloxidases, are enzymes involved in the oxidation of phenolic compounds. Tyrosinases catalyse the *o*-hydroxylation of monophenols and oxidation of *o*-diphenols to *o*-quinones [9,10], whereas catechol oxidases are only able to catalyse the oxidation of *o*-diphenols [11]. Hemocyanins are mainly oxygen carrier proteins that under specific circumstances have enzymatic activity [6,10,12-14]. All type-3 copper proteins share a similar binuclear active site that is composed of two copper atoms [Cu(A) and Cu(B)], each of which is coordinated by three conserved histidine residues [11,15]. Although type-3 copper proteins possess similar active sites in terms of overall structure and ability to bind molecular oxygen, they each differ in amino acid sequence [6,10,15,16]. These amino acid differences affect the substrate-binding pocket and the accessibility of the substrate to the active site [15].

The differences in the active site of type-3 copper proteins are likely to have enabled the diversification of these proteins into a wide array of biological processes, including oxygen transport in molluscs and arthropods, innate immunity, wound healing and melanin pigment synthesis in a variety of metazoans, and the browning of fruits and vegetables [5,10,17-19]. Some of the chemical reactions catalysed by catechol oxidases and tyrosinases are thought to protect plants and animals by the formation of melanised structures that can encase foreign invaders [5,19]. Tyrosinases also contribute to calcified structures, such as molluscan shells and arthropod exoskeletons [20-23].

Despite the broad distribution and importance of type-3 copper proteins, the evolution of this family has only been studied in detail in plants (catechol oxidases) and specific animal lineages, including chordates (tyrosinases and tyrosinase related-proteins), arthropods and molluscs (tyrosinases and hemocyanins) [10,16,24-31]. Molluscs and arthropods both possess tyrosinases and hemocyanins, however previous studies have shown that tyrosinases and hemocyanins differ considerably at the amino acid level between these organisms, indicating that both proteins evolved independently [16,25,26,32,33]. In chordates, gene structure and phylogenetic analyses suggest that tyrosinases arose from a single ancestral gene [24,28,34] that duplicated before the divergence of urochordate and vertebrate lineages [35], leading to genes encoding tyrosinase and tyrosinase related-proteins. The latter then duplicated early in vertebrate evolution, giving rise to tyrosinase related-proteins 1 and 2 [24,27,30]. In chordates, tyrosinase related-proteins are grouped under the tyrosinase gene family, although not all these genes have tyrosinase activity (e.g., tyrosinase related-protein 2 has dopachrome tautomerase activity and there is some evidence that it binds zinc rather than copper at the active site) [36]. In plants, catechol oxidase genes display a dynamic and complex evolutionary pattern (e.g. soy bean has undergone extensive species-specific gene duplication and divergence) [31]. Here, we analyse available genomic resources of diverse metazoan and other eukaryotic and prokaryotic species to reconstruct the origin and evolution of type-3 copper proteins and propose a new classification scheme for this protein family.

## Results

### Division of type-3 copper proteins into three subclasses based on domain architecture and conserved residues in the copper-binding sites

A systematic search of sequenced genomes revealed the presence of type-3 copper proteins in representatives of the three domains of cellular life (see Additional file 1 for accession numbers, genome localisation, protein nomenclature used in this study and phylogenetic group of the representatives of each domain of life). No type-3 copper proteins were detected in the draft genomes of the sea urchin *Strongylocentrotus purpuratus*, the placozoan *Trichoplax adhaerens*, the choanoflagellate *Monosiga brevicolis* and the filose amoeboid holozoan *Capsaspora owczarzaki*.

**Figure 1:**
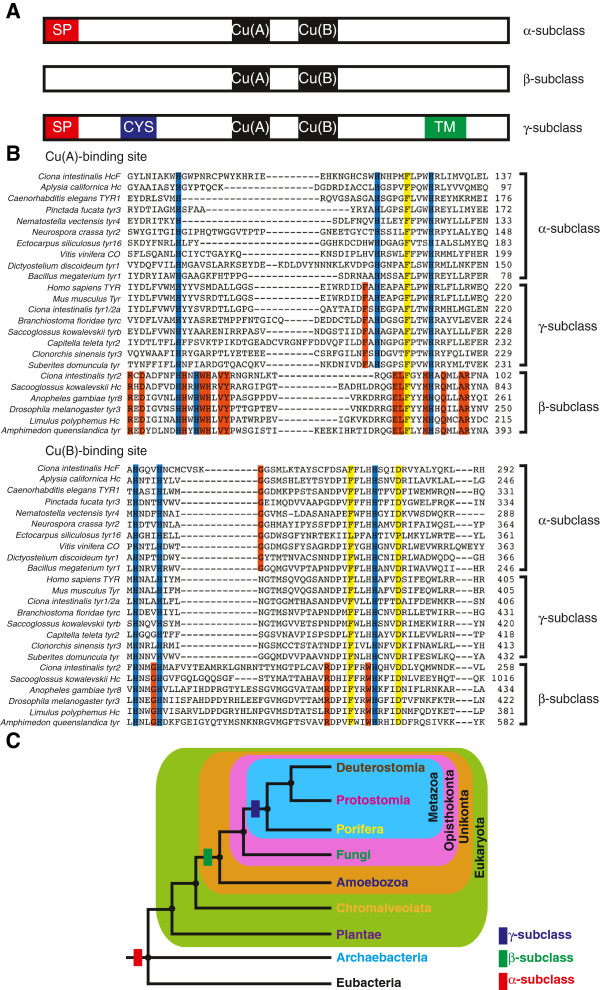
**Domain architecture, copper-binding site alignment and origin of type-3 copper protein subclasses. A**. Schematic representation of domain architecture of each type-3 copper protein subclass. SP: signal peptide; CYS: cysteine-rich regions; Cu(A) and Cu(B): copper-binding sites; TM: transmembrane region. **B**. Sequence alignment of both copper-binding sites from representatives of each type-3 copper protein subclass. Active-site histidine residues, which are important in copper-binding site conformation (blue), and conserved amino acids across all subclasses (yellow), as well as conserved amino acids restricted to specific subclasses (orange) that might have important role in structural conformation. See Additional file 1 for protein nomenclature. **C**. A simplified phylogenetic tree of the three domains of life showing the emergence of α-, β- and γ-subclasses.

All type-3 copper proteins possess a conserved pair of copper-binding sites, called Cu(A) and Cu(B), however they can be further categorised into subclasses based on the possession of other conserved domains or motifs. One subclass (α) has an N-terminal signal peptide, indicative of being secreted or localised to vesicles, another subclass (β) lacks this domain and appears to be localised to the cytosol, and the third subclass (γ) possesses an N-terminal signal peptide, a cysteine-rich region, which may be involved in protein-protein interactions or dimerization, and a transmembrane domain consistent with it being a membrane-bound form (Figure 1A).

The Cu(A)-binding site is characterised by a H_1_(*n*)-H_2_(8)-H_3_ motif and the Cu(B)-binding site by a H_1_(3)-H_2_(*n*)-H_3_ motif, where *n* is a variable number of residues between histidines. This histidine arrangement is conserved in α- and γ-subclass type-3 copper proteins, whilst β-subclass type-3 copper proteins exhibit a different position of the second histidine residue in the Cu(A)-binding site (Figure 1B). There are several amino acids that are conserved across all copper protein subclasses, (e.g. Phe four residues upstream from H_3_ in both copper-binding sites and Asp four residues downstream from H_3_ of the Cu(B)-binding site) and there are a number of diagnostic residues restricted to each subclass (Figure 1B). Conserved sites are potentially important for the structural conformation of these proteins, whilst differences may affect substrate preferences.

**Figure 2:**
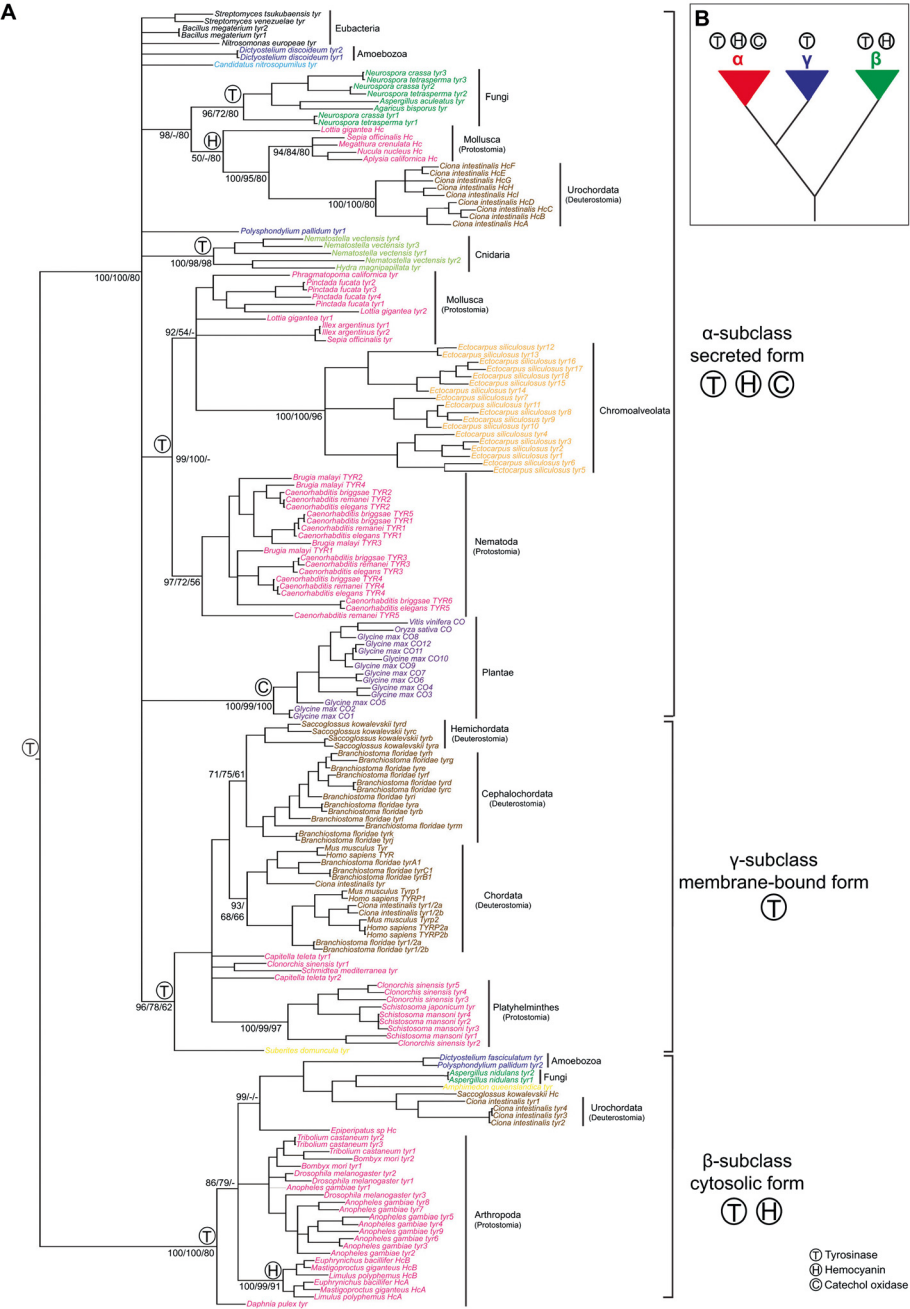
**Phylogenetic analysis of the type-3 copper subclass proteins. A**. A representative phylogenetic tree based on Bayesian Inference (BI), which is midpoint rooted. Statistical support is indicated at the nodes; first number, BI posterior probabilities; second number, ML bootstrap support; third number, NJ bootstrap support. Only statistical support values >50% are shown. Accession numbers of the proteins used in this tree can be found in Additional file 1. See Additional files 2, 3, 4 for detailed phylogenetic analyses of each type-3 copper protein subclass. **B**. Phylogenetic relationship and functionalities found in type-3 copper subclasses. T, tyrosinase; C, catechol oxidases; H, hemocyanin. Species were labelled according to a specific colour code as follows: black: Eubacteria; sky blue: Archaeobacteria; purple: Plantae; orange: Chromoalveaolata; dark blue: Amoebozoa; dark green: Fungi; yellow: Porifiera; light green: Cnidaria; magenta: Protostomia (Mollusca, Annelida, Platyhelminthes, Nematoda, Arthropoda and Onycophora); brown: Deuterostomia (Hemichordata, Cephalochordata, Urochordata and Vertebrata).

The Metazoa is the only kingdom to possess all three subclasses of type-3 copper proteins. All surveyed non-holozoan unikonts (e.g., amoebozoans and fungi) lack members of the γ-subclass. Only the α-subclass is present in the non-unikont eukaryote, bacterial and archaeal genomes examined. This phylogenetic distribution of type-3 copper protein subclasses is consistent with the secreted α-subclass being ancestral and potentially present in the last universal common ancestor to all cellular life (Figure 1C). This ancestral type-3 copper protein likely duplicated and diverged along the unikont stem prior to the divergence of amoebozoan and opisthokont lineages, giving rise to the cytosol-localised β-subclass. As the membrane-bound γ form is present only in metazoan genomes (no type-3 copper proteins were detected in the holozoans *Monosiga brevicolis* and *Capsaspora owczarzaki*) and is more closely related to α-subclass type-3 copper proteins (Figures 1B and 2A), we infer that the γ-subclass arose from a second duplication of an α-subclass type-3 copper protein gene.

### Phylogenetic analyses support the three subclasses of type-3 copper proteins and reveal multiple lineage-specific expansions of specific subclasses

The three subclasses of type-3 copper proteins identified above were supported by phylogenetic analyses, with the β-subclass being the most divergent (Figure 2A). Our phylogenetic analysis positions the γ-subclass as a clade within the unresolved α-subclass polytomy. This membrane bound form of the type-3 copper proteins includes only tyrosinases and tyrosinase related-proteins (Figure 2).

Based on previous structural and enzymatic analyses (e.g., [15,37-46]), the α-subclass includes tyrosinases, catechol oxidases and molluscan and urochordate hemocyanins. Our phylogenetic analyses reveal that the α-subclass is not present in representatives of multiple metazoan phyla and subphyla, including poriferans, placozoans, platyhelminths, arthropods, hemichordates, echinoderms, cephalochordates and chordates (Figure 2 and Additional file 2). Molluscan and urochordate hemocyanins are within the α-subclass and form a well-supported monophyletic group, except for the *Lottia gigantea* hemocyanin (Figure 2A). This is consistent with either the last common ancestor to extant bilaterians having an α-subclass hemocyanin, which has been lost subsequently in most phyla, or the convergent evolution of this gene in molluscs and urochordates.

In each of these subclasses, there are cases of lineage-specific gene expansions. Figure 2A and Additional files 2, 3, 4 display phylogenetic relationships of each type-3 copper protein subclass where there are examples of lineage-specific gene expansions, as is the case in the soy bean (α-subclass), nematodes (α-subclass), mosquitoes (β-subclass), and amphioxus (γ-subclass). Generally, this has resulted in the expansion of one functional class of type-3 copper protein, such the 18 tyrosinases in amphioxus. In arthropods, duplication of β-subclass type-3 copper proteins gave rise to contemporary arthropod hemocyanin and tyrosinase proteins (Figure 2A; Additional file 3).

Analysis of the genomic structure of type-3 copper protein genes show that soy bean (*Glycine max*, α-subclass), brown algae (*Ectocarpus siliculosus*, α-subclass), mosquito (*Anopheles gambiae* β-subclass), and amphioxus (*Branchiostoma floridae* γ-subclass) all possess clusters of two to seven linked genes in their genomes (Additional file 5), consistent with subclass expansions largely being the result of tandem gene duplication events.

### Structural changes at the binuclear copper active site underlie the evolution of tyrosinase, catechol oxidase and hemocyanin functionalities

**Figure 3:**
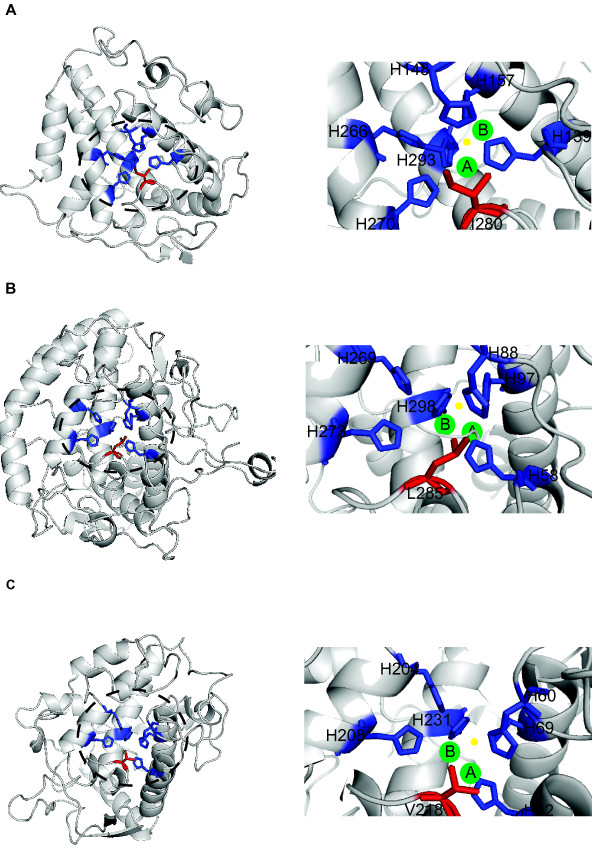
**Binuclear active site of tyrosinase proteins and placeholder residue blocking the entrance of the substrate.** Stereo view of tyrosinase active site region with both Cu-binding sites is presented. Copper ions are depicted in green, Cu(A) is shown on the left and Cu(B) is shown on the right. The yellow sphere represents a dioxygen molecule. In each structure, the occupation and positioning of copper varies. The six copper-coordinating histidine ligands coordinating the structural conformation of the active site are shown in dark blue. In addition, the placeholder residue that reaches into the active site above Cu(A) and blocks the substrate-binding pocket and the entrance of the substrate into the active site is indicated in red. Differences in the orientation and size of the blocking residue are key to the enzymatic activity of tyrosinases. Representatives of three tyrosinase proteins from α- subclass are shown. **A**. Mollusc tyrosinase, **B**. Nematode tyrosinase and **C**. Cnidarian tyrosinase.

Homology modelling of type-3 copper proteins based on published crystal structures [38-41,45,46] and pairwise comparisons provides insight into the functional constraints on amino acids associated with the active site and how steric effects might underlie differences in molecular oxygen binding and the enzymatic activity of tyrosinases, tyrosinase-related proteins, catechol oxidases and hemocyanins. Comparison between all type-3 copper protein subclasses reveals a set of key residues around the active site that are specific to each subclass (Figure 1B and Table 1). Although the precise functionality of these residues is currently unknown, it is probable that these residues are associated with a specific type-3 copper protein function. One of these residues appears to act as a placeholder for phenolic substrates, stabilising the active site geometry and the binding and docking of the substrate to the Cu(A)-binding active site (Table 1) [37-46]. This placeholder residue varies, depending on the species and type-3 copper protein subclass. The α-subclass proteins display a spectrum of placeholder residues, including hydrophobic and aromatic amino acids, whereas β- and γ-subclass proteins possess Phe and Val residues, respectively (Figure 3 and Table 1). An interesting feature present in some members of type-3 copper proteins is the presence of a covalent bond between a cysteine residue and the second histidine of the Cu(A)-binding site [45,46]. Cysteine-histidine bonds have been proposed to be involved in electron transfer, however the function of this bond in the enzymatic mechanisms of type-3 copper proteins remain unclear. Disulfide bridges may be involved in the stabilisation of the active site and/or protein folding

**Table 1:** Structural differences in the active site of type-3 copper proteins

	**Place-holder residue**^**1**^	**Cysteine-histidine bound**^**2**^	**Disulfide bridges**^**3**^
**α-subclass protein**			
Eubacteria tyrosinases	V	No	No
Archaebacteria tyrosinases	V	No	No
Plantae catechol oxidases	F/L	Yes	2 bridges
Chromalveolata tyrosinases	V	No	No
Amoebozoan tyrosinases	V	No	No
Fungal tyrosinases	V/L/I	Yes	No
Cnidarian tyrosinases	V	No	No
Molluscan hemocyanins	L	Yes	2 bridges
Molluscan tyrosinases	V/I	No	No
Nematode tyrosinases	I	No	No
Urochordate hemocyanins	L	Yes	2 bridges
**β-subclass protein**			
Amoebozoan tyrosinases	F	No	No
Fungal tyrosinases	F	No	No
Poriferan tyrosinase	F	No	1 bridge
Arthropod tyrosinases	F	No	2 bridges
Arthropod hemocyanins	F	No	2 bridges
Hemichordate hemocyanin	F	No	No
Urochordate tyrosinases	F	No	No
**γ-subclass protein**			
Poriferan tyrosinase	V	No	No
Platyhelminth tyrosinases	V	No	No
Annelid tyrosinases	V	No	No
Hemichordate tyrosinases	V	No	No
Cephalochordate tyrosinases	V	No	No
Urochordate tyrosinases	V	No	No
Vertebrate tyrosinases	V	No	No

^1^The placeholder residue blocks the access of the substrate to the active site, affecting the enzymatic activity of type-3 copper proteins.

^2^Cysteine-histidine bond is proposed to be involved in the electron transfer process; however, its function is still contentious.

^3^Disulfide bridges stabilise protein folding.

## Discussion

The type-3 copper protein family is ancient, apparently antedating the divergence of the three domains of life. This antiquity, along with diversity of functions observed in extant members, has made it difficult to determine the ancestral function and early evolution of this family. By placing genomic and structural data in a phylogenetic framework, we have reconstructed the evolution of this protein family and demonstrated that cytosolic (β) and membrane-bound (γ) forms evolved from an ancestral type-3 copper protein that was probably secreted (α). Subtle changes in the amino acid composition of the binuclear copper active site led to the differing functionalities present in extant hemocyanins, catechol oxidases, tyrosinases and tyrosinase-related proteins.

### Evolutionary history of type-3 copper genes is characterised by lineage-specific gene expansions and losses

**Figure 4:**
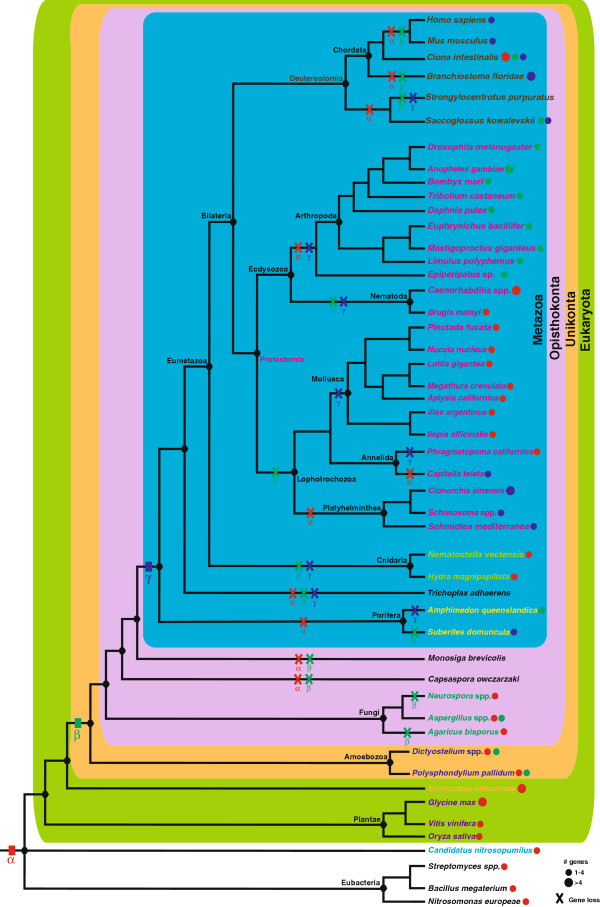
**Summary of gene expansions and losses of type-3 copper protein subclasses.** The phylogenetic relationships between the species under study are shown. The ancestral form (α-subclass, red colour code) arose early in the evolution of life. The β-subclass (green colour code) emerged before the divergence of unikont lineages. Finally, a γ-subclass (blue colour code) emerged as a second duplication of the α-subclass ancestor prior to metazoan diversification. Coloured crosses denote cases for which losses of particular subclasses have occurred in specific lineages. Gene expansions are indicated by coloured dots for each lineage. Species are labelled according to colour code shown in Figure 2.

We identified 179 type-3 copper proteins in 35 metazoan and 17 non-metazoan species. Phylogenetic analyses reveal that in addition to the sequential evolution of α-, β- and γ-subclasses there have been multiple, independent gene loss and expansion events often resulting in lineage-specific paralogy groups. Gene loss is a common feature during the evolutionary history of type-3 copper proteins with some subclasses being present only in specific metazoan phyla (e.g., cnidarians, platyhelminthes, molluscs, arthropods, cephalochordates and vertebrates). Gene loss appears to have been so extensive that we were only able to detect one species, the urochordate *Ciona intestinalis*, possessing all three subclasses (Figure 4 and Additional file 1). Indeed, no genes encoding type-3 copper proteins were detected in the draft genomes of *Strongylocentrotus purpuratus* (sea urchin), *Trichoplax adhaerens* (placozoan), *Monosiga brevicolis* (choanoflagellate) and *Capsaspora owczarzaki* (filose amoeboid holozoan). The absence of type-3 copper proteins suggests that these species possess different protein repertories for the oxidation of phenolic compounds or oxygen transport. In most species, the diversification of type-3 copper proteins is largely a consequence of lineage-specific expansion of one or two subclasses. This appears to be the primary mechanism by which most species acquire multiple type-3 copper proteins. For example, α-subclass type-3 copper protein in plants (*G. max*, 12 genes), chromalveolates (*E. siliculosus*, 18 genes) and nematodes (*C. elegans*, 5 genes; *C. briggsae*, 6 genes; *C. remanei*, 5 genes and *B. malayi*, 4 genes) have undergone large gene expansions, whilst β- and γ- subclasses have expanded significantly in arthropods (*A. gambiae*, 9 genes) and cephalochordates (*B. floridae*, 18 genes).

In most cases, the functions of duplicated type-3 copper protein genes are unknown. New catalytic activities and metal binding properties may have evolved, as is the case of the tyrosinase-related protein 2, which uses zinc instead of copper as cofactor. Despite this difference, its binding to the active sites is coordinated by three conserved histidine residues, [36]. In vertebrates, multiple tyrosinase and tyrosinase-related proteins are involved in a complex and tightly regulated process of pigmentation. The complexity is in stark contrast to pigmentation in bacteria, plants and non-chordate invertebrates, which is a single step process where melanogenesis is enzymatically controlled by a small number of tyrosinase proteins [30,36]. Thus, lineage-specific gene duplication events are likely to lead to expansion in functionality of type-3 copper proteins in specific taxa.

Co-option of new paralogues into novel functions (neofunctionalisation) is a common outcome of gene duplication events [47] and may underlie adaptations to specific ecological niches [48-51]. The evolution (in two independent events) of hemocyanins as specialised oxygen-transporting proteins can be viewed as an example of neofunctionalisation within type-3 copper binding proteins; more examples of neofunctionalisation within this family may come to light as the functions of the proteins are discovered. It is also possible that subfunctionalisation is a key driving force for the retention of multiple family members. A multitude of functions have been ascribed to type-3 copper binding proteins, therefore gene duplication and retention may represent a partitioning of these functions between a number of genes.

### Structural comparison of type-3 copper protein active sites and insights into enzymatic mechanisms

All type-3 copper protein family members reversibly bind dioxygen, whilst only catechol oxidases and tyrosinases oxidise diphenols and only tyrosinases oxygenate monophenols [52]. The enzymatic mechanism of type-3 copper proteins is still a subject of debate due to the existence of two catalytic activities at the same active site (monophenolase and diphenolase activitites) and the ability to transport dioxygen. In this study, homology modelling of the active site of each type-3 copper protein subclass reveals a conserved hydrophobic core, comprising a four-helix bundle with two histidine-coordinated copper atoms, Cu(A) and Cu(B). This structural conformation of the active site is highly conserved among catechol oxidases, tyrosinases, tyrosinase-related proteins and hemocyanins [38-46].

Access to the active site is blocked by different placeholder amino acids. These blocking residues appear to affect the ability to bind molecular oxygen as well as the enzymatic activity of type-3 copper proteins [15,39]. For instance, catechol oxidases lack monophenolase activity due to the presence of the bulky placeholder amino acid phenylalanine that is located near to the Cu(A)-binding site, blocking the entrance of monophenol substrates [39]. In tyrosinases, either a valine or isoleucine residue acts as placeholder. Both small amino acids allow the docking of monophenol substrates to the Cu(A)-binding site and diphenol substrates to the Cu(B)-binding site, allowing tyrosinases to oxidise both monophenol and diphenol substrates [36,38,44]. In the active site of hemocyanins, there is not enough space for phenolic substrate binding because of the complex organisation of hemocyanins into subunits and domains [53]. This structural organisation only enables the reversible binding of small molecules such as dioxygen, thus these proteins as storage and carrier proteins in molluscs, arthropods and ascidians [6,16].

Previous phylogenetic reconstructions between mollusc and arthropod type-3 copper genes have indicated that these proteins have evolved independently [10,16,25,26,54]. Here, we demonstrate that mollusc and arthropod type-3 copper genes belong to different subclasses (α- and β-subclass, respectively), supporting previous assertions of their independent origin and convergent evolution. The emergence of hemocyanin respiratory proteins in arthropods and molluscs, and possibly urochordates, is postulated to have occurred independently during the Neoproterozoic with an increase in atmospheric oxygen and animal body size [55]. The range of oxygen-transport proteins present in contemporary animals [56] is consistent with respiratory proteins evolving from a range of ancestral proteins, including α- and β-subclasses of type-3 copper proteins identified in this study. In summary, primitive type-3 copper proteins, probably tyrosinases (Figure 2A), are likely to have exhibited broad activities and/or substrate affinities, and subsequent fine-tuning of the active site enabled the evolution of the different functionalities of catechol oxidases, tyrosinases, tyrosinase related-proteins and hemocyanins in extant animals.

## Conclusion

We have classified type-3 copper proteins into three subclasses based on domain architecture differences, phylogenetics and the presence and absence of these forms in the genomes of extant organisms representing disparate lineages of cellular life. Specifically, we postulate that an ancestral secreted form (α-subclass) of type-3 copper protein emerged early in the evolution of life. This ancestral gene duplicated and diverged prior to the split of unikont eukaryote lineages, giving rise to a cytoplasmic β-subclass type-3 copper protein. The membrane-bound γ-subclass is likely to have arisen as a result of a second duplication of the α-subclass gene before metazoan diversification.

Our analyses demonstrate that type-3 copper protein family is evolutionarily dynamic and characterised by multiple and independent lineage-specific gene expansions and differential gene losses of one or more subclasses. This complex evolutionary history likely reflects the diversity of type-3 copper functions observed in contemporary animals, which may be related to their different lifestyles, specific adaptations and degree of speciation of these phyla.

## Methods

### Retrieval of type-3 copper sequences

A systematic search for type-3 copper proteins was conducted by the BLASTP algorithm [57] using a threshold e-value of 1e-5. Type-3 copper protein sequences from mouse (*Mus musculus*; accession number: NP_035791.1), fruit fly (*Drosophila melanogaster*; accession number: NP_524760.1), keyhole limpet (*Megathura crenulata*; accession number: CAG28310.1) and rice (*Oryza sativa*; accession number: NP_001053932.1) were used as queries. Protein sequences having InterProScan and/or PFAM protein domains corresponding to tyrosinase (IPR002227 and PF00264) and hemocyanin (IPR005204 and PF03723) were kept for further analyses. The analysed databases included NCBI non-redundant database and the current versions of predicted proteins of the genome of *Branchiostoma floridae* (http://genome.jgi.doe.gov/Brafl1/Brafl1.home.html), *Ciona intestinalis* (http://genome.jgi.doe.gov/Cioin2/Cioin2.info.html), *Strongylocentrotus purpuratus* (http://www.metazome.net/search.php), *Lottia gigantea* (http://genome.jgi.doe.gov/Lotgi1/Lotgi1.info.html), *Capitella teleta* (http://genome.jgi.doe.gov/Capca1/Capca1.home.html), *Nematostella vectensis* (http://genome.jgi.doe.gov/Nemve1/Nemve1.info.html)*, Trichoplax adhaerens* (http://genome.jgi.doe.gov/Triad1/Triad1.info.html), *Amphimedon queenslandica* (http://spongezome.metazome.net/cgi-bin/gbrowse/amphimedon/)*, Monosiga brevicolis* (http://genome.jgi.doe.gov/Monbr1/Monbr1.home.html)*, Capsaspora owczarzaki* (http://www.broadinstitute.org/annotation/genome/multicellularity_project/GenomeDescriptions.html)*, Neurospora tetrasperma* (http://genome.jgi.doe.gov/Neute_matA2/Neute_matA2.home.html)*, Aspergillus aculeatus* (http://genome.jgi.doe.gov/Aspac1/Aspac1.home.html), and *Ectocarpus siliculosus* (http://bioinformatics.psb.ugent.be/webtools/bogas/overview/Ectsi).

### Phylogenetic analyses

Multiple alignments of full-length proteins were carried out using the MAFFT algorithm [58] and edited manually using Geneious v5.1.7 (Biomatters Ltd.). The alignments were analysed with Gblocks 0.91b software [59] using default parameters, to select conserved regions. The best amino acid substitution model was chosen using ProtTest v3.0 [60].

Neighbor-Joining (NJ) reconstructions were performed using MEGA 5.05 [61] with 1,000 bootstrap replicates. Maximum Likelihood (ML) analyses were conducted by PhyML 3.0 [62]. Statistical support for the different internal branches was assessed through bootstrap resampling (1,000 replicates). Bayesian Inference (BI) was performed using the Markov chain Monte Carlo method as implemented in MrBayes v3.2 [63]. Two independent runs were performed, each containing 4 Markov chains and 2,500,000 to 10,000,000 generations. One out of every 1,000 trees was saved. The trees obtained in the two runs were meshed and the first 25% of the trees were discarded as ‘burnin’. Marginal probabilities at each internal branch were taken as a measure of statistical support. All phylogenetic trees were visualized and edited using FigTree v1.3.1 (http://tree.bio.ed.ac.uk/software/figtree/). Alignments and phylogenies were submitted to treeBASE database under study ID 13985 (Access at: http://treebase.org/treebase-web/search/study/summary.html?id=13985).

### Domain architecture analysis and secondary structure prediction

Domain architecture was determined using InterProScan and PFAM databases [64]. Putative signal peptides were identified using the SignalP v4.0 and ChloroP v1.1 Servers [65,66]. Transmembrane regions were detected using TMHMM Server v2.0 (http://www.cbs.dtu.dk/services/TMHMM/).

To identify functional amino acids around the active site, 3D structures of each type-3 copper protein were reconstructed using the Phyre2 Server [67]. Homology modelling was performed using representatives of type-3 copper proteins with known 3D structure: [PDB: 1BT1] plant catechol oxidase (*Ipomoea batatas*) [39]; [PDB: 1OXY] arthropod hemocyanin (*Limulus polyphemus*) [41]; [PDB: 1JS8] molluscan hemocyanin (*Octopus dofleini*) [46]; [PDB: 3HHS] arthropod phenoloxidase (*Manduca sexta*) [40]; [PDB: 2Y9W] fungal tyrosinase (*Agaricus bisporus*) [45]; and [PDB: 3NM8] bacterial tyrosinase (*Bacillus megaterium*) [38]. Protein tertiary structure alignments were conducted by the Calculate Structure Alignment software using the jCE algorithm and default parameters [68]. All protein structure images were generated using PyMOL (http://www.pymol.org/).

## Competing interests

The authors declare that they have no competing interests.

## Authors’ contributions

FA obtained the data and performed phylogenetic, sequence and prediction of secondary structure analyses, participated in the design of the study and drafted the manuscript. FA, CM and BMD conceived the study, participated in its design, were involved in data analyses and drafting of the manuscript. All authors have read and approved the final manuscript.

## Supplementary Material

Additional file 1 **Dataset: List of accession numbers, genome localisation and protein nomenclature used in this study.** Accession numbers from Genbank and genomic localisations from Joint Genome Institute (JGI) and Bioinformatics Online Genome Annotation Services (BOGAS) for type-3 copper protein sequences. Click here for file

Additional file 2 **Phylogenetic trees: Phylogenetic analyses of the α-subclass copper proteins.** A. Neighbor-Joining (NJ) phylogenetic tree is shown. Statistical support for each node is indicated as percentage (1,000 bootstrap reanalyses). B. Maximum-Likelihood (ML) phylogenetic tree is shown. Statistical support for each node is indicated as percentage (1,000 bootstrap reanalyses). C. Bayesian Inference (BI) phylogenetic tree is shown. Statistical support for each node is indicated as posterior probabilities (2,500,000 generations). In all cases, trees were rooted by midpoint rooting and labelled as in Figure 2A. Click here for file

Additional file 3 **Phylogenetic trees: Phylogenetic analyses of the β-subclass copper proteins.** A. Neighbor-Joining (NJ) phylogenetic tree is shown. Statistical support for each node is indicated as percentage (1,000 bootstrap reanalyses). B. Maximum-Likelihood (ML) phylogenetic tree is shown. Statistical support for each node is indicated as percentage (1,000 bootstrap reanalyses). C. Bayesian Inference (BI) phylogenetic tree is shown. Statistical support is indicated as posterior probabilities (2,500,000 generations). In all cases, trees were rooted by midpoint rooting and labelled as in Figure 2A. Click here for file

Additional file 4 **Phylogenetic trees: Phylogenetic analyses of the γ-subclass copper proteins.** Neighbor-Joining (NJ) phylogenetic tree is shown. Statistical support for each node is indicated as percentage (1,000 bootstrap reanalyses). B. Maximum-Likelihood (ML) phylogenetic tree is shown. Statistical support for each node is indicated as percentage (1,000 bootstrap reanalyses). C. Bayesian Inference (BI) phylogenetic tree is shown. Statistical support is indicated as posterior probabilities (2,500,000 generations). In all cases, trees were rooted by midpoint rooting and labelled as in Figure 2A. Click here for file

Additional file 5 **Lineage-specific expansion of type-3 copper gene subclasses and their physical linkage: Putative physical linkages of some representatives from α-, β-, and γ-subclasses.** Type-3 copper genes that are physically linked. Exons are indicated by boxes; while introns are indicated by lines adjoining these. Copper-binding sites A and B are indicated by black boxes Cu(A) at the left and Cu(B) at the right, respectively. Intergenic distances are indicated in kilobases. All gene structures are drawn to scale but these scales differ between phyla and subclasses. The arrow indicates the direction of transcription for each gene. Click here for file
