# Supplementary material for: Origin, evolution and classification of type-3 copper proteins: lineage-specific gene expansions and losses across the Metazoa
Source: BMC Evol Biol. 2013 May 1;13:96. doi: 10.1186/1471-2148-13-96 (PMC3658974; doi:10.1186/1471-2148-13-96)
Supplement: Additional file 2 — Phylogenetic trees: Phylogenetic analyses of the α-subclass copper proteins. A. Neighbor-Joining (NJ) phylogenetic tree is shown. Statistical support for each node is indicated as percentage (1,000 bootstrap reanalyses). B. Maximum-Likelihood (ML) phylogenetic tree is shown. Statistical support for each node is indicated as percentage (1,000 bootstrap reanalyses). C. Bayesian Inference (BI) phylogenetic tree is shown. Statistical support for each node is indicated as posterior probabilities (2,500,000 generations). In all cases, trees were rooted by midpoint rooting and labelled as in Figure 2A. [file 1471-2148-13-96-S2.pdf]

A

α-subclass phylogenetic tree obtained by Neighbor-Joining method

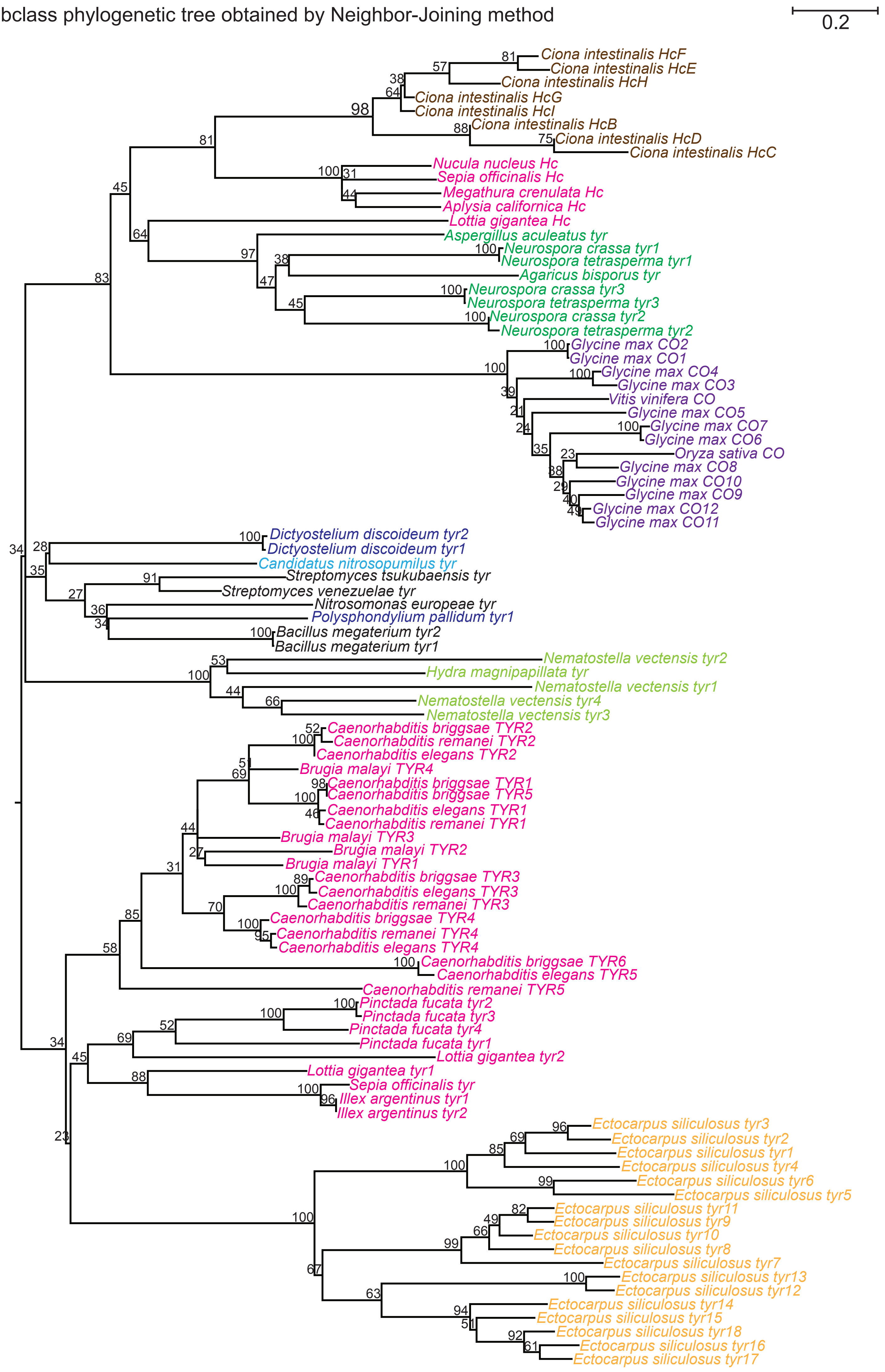

B

α-subclass phylogenetic tree obtained by Maximum-Likelihood method

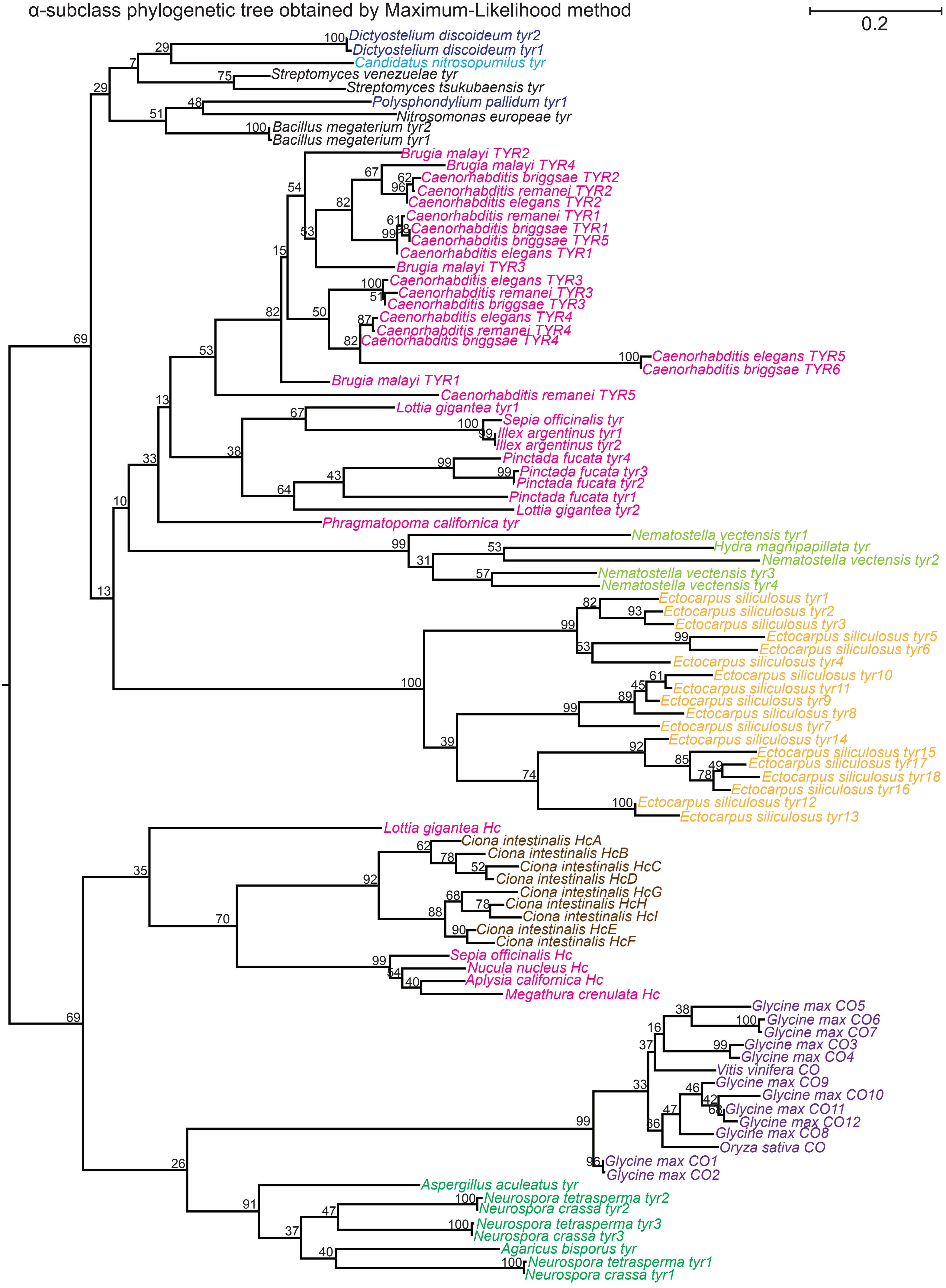

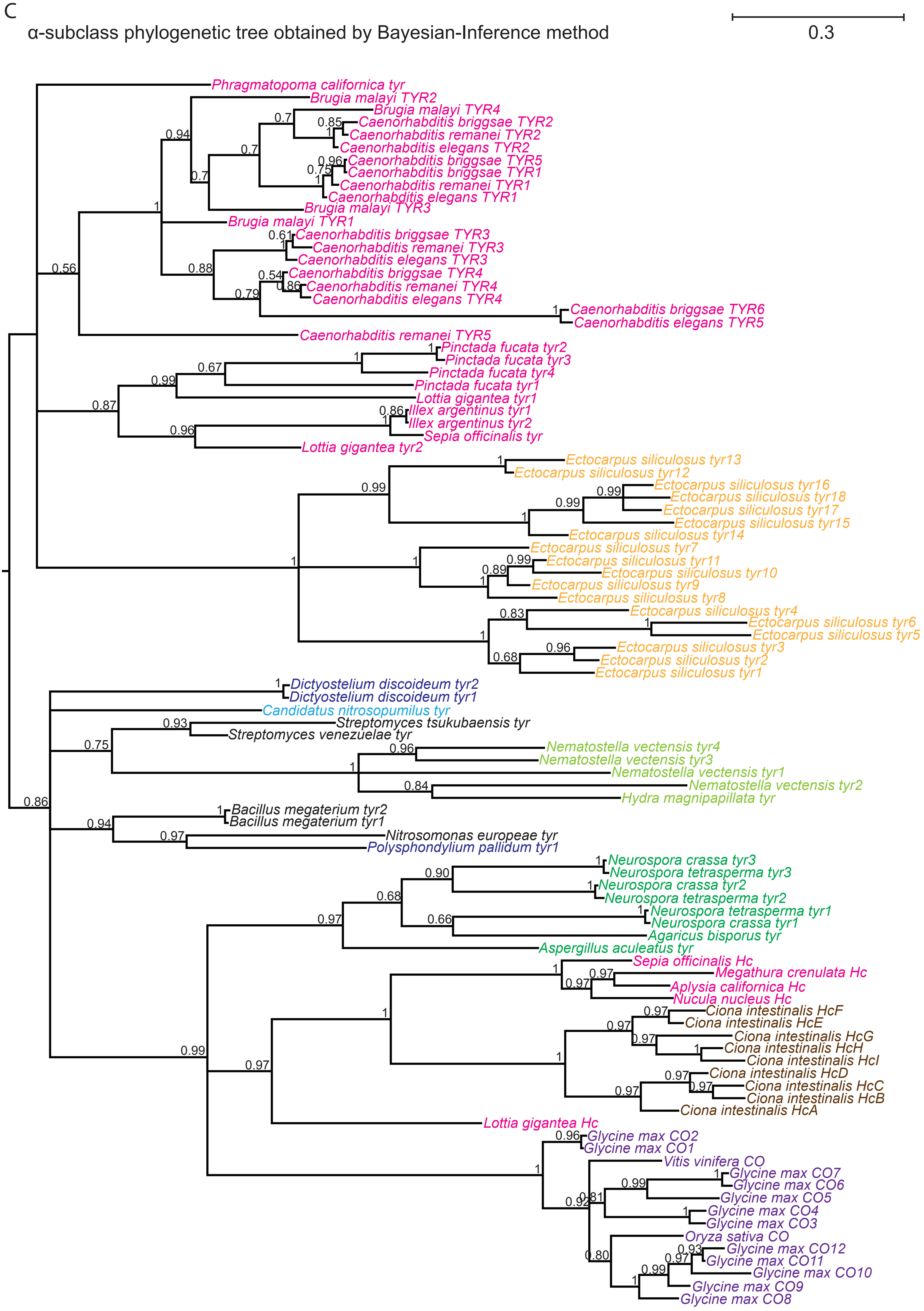
C
α-subclass phylogenetic tree obtained by Bayesian-Inference method
0.3
Phragmatopoma californica tyr
Brugia malayi TYR2
Brugia malayi TYR4
Caenorhabditis briggsae TYR2
Caenorhabditis remanei TYR2
Caenorhabditis elegans TYR2
Caenorhabditis briggsae TYR5
Caenorhabditis briggsae TYR1
Caenorhabditis remanei TYR1
Caenorhabditis elegans TYR1
Brugia malayi TYR3
Brugia malayi TYR1
Caenorhabditis briggsae TYR3
Caenorhabditis remanei TYR3
Caenorhabditis elegans TYR3
Caenorhabditis briggsae TYR4
Caenorhabditis remanei TYR4
Caenorhabditis elegans TYR4
Caenorhabditis briggsae TYR6
Caenorhabditis elegans TYR5
Caenorhabditis remanei TYR5
Pinctada fucata tyr2
Pinctada fucata tyr3
Pinctada fucata tyr4
Pinctada fucata tyr1
Lottia gigantea tyr1
Illex argentinus tyr1
Illex argentinus tyr2
Sepia officinalis tyr
Lottia gigantea tyr2
Ectocarpus siliculosus tyr13
Ectocarpus siliculosus tyr12
Ectocarpus siliculosus tyr16
Ectocarpus siliculosus tyr18
Ectocarpus siliculosus tyr17
Ectocarpus siliculosus tyr15
Ectocarpus siliculosus tyr14
Ectocarpus siliculosus tyr7
Ectocarpus siliculosus tyr11
Ectocarpus siliculosus tyr10
Ectocarpus siliculosus tyr9
Ectocarpus siliculosus tyr8
Ectocarpus siliculosus tyr4
Ectocarpus siliculosus tyr6
Ectocarpus siliculosus tyr5
Ectocarpus siliculosus tyr3
Ectocarpus siliculosus tyr2
Ectocarpus siliculosus tyr1
Dictyostelium discoideum tyr2
Dictyostelium discoideum tyr1
Candidatus nitrosopumilus tyr
Streptomyces tsukubaensis tyr
Streptomyces venezuelae tyr
Nematostella vectensis tyr4
Nematostella vectensis tyr3
Nematostella vectensis tyr1
Nematostella vectensis tyr2
Hydra magnipapillata tyr
Bacillus megaterium tyr2
Bacillus megaterium tyr1
Nitrosomonas europeae tyr
Polysphondylium pallidum tyr1
Neurospora crassa tyr3
Neurospora tetrasperma tyr3
Neurospora crassa tyr2
Neurospora tetrasperma tyr2
Neurospora tetrasperma tyr1
Neurospora crassa tyr1
Agaricus bisporus tyr
Aspergillus aculeatus tyr
Sepia officinalis Hc
Megathura crenulata Hc
Aplysia californica Hc
Nucula nucleus Hc
Ciona intestinalis HcF
Ciona intestinalis HcE
Ciona intestinalis HcG
Ciona intestinalis HcH
Ciona intestinalis HcI
Ciona intestinalis HcD
Ciona intestinalis HcC
Ciona intestinalis HcB
Ciona intestinalis HcA
Lottia gigantea Hc
Glycine max CO2
Glycine max CO1
Vitis vinifera CO
Glycine max CO7
Glycine max CO6
Glycine max CO5
Glycine max CO4
Glycine max CO3
Oryza sativa CO
Glycine max CO12
Glycine max CO11
Glycine max CO10
Glycine max CO9
Glycine max CO8
