# Supplementary material for: Origin, evolution and classification of type-3 copper proteins: lineage-specific gene expansions and losses across the Metazoa
Source: BMC Evol Biol. 2013 May 1;13:96. doi: 10.1186/1471-2148-13-96 (PMC3658974; doi:10.1186/1471-2148-13-96)
Supplement: Additional file 3 — Phylogenetic trees: Phylogenetic analyses of the β-subclass copper proteins. A. Neighbor-Joining (NJ) phylogenetic tree is shown. Statistical support for each node is indicated as percentage (1,000 bootstrap reanalyses). B. Maximum-Likelihood (ML) phylogenetic tree is shown. Statistical support for each node is indicated as percentage (1,000 bootstrap reanalyses). C. Bayesian Inference (BI) phylogenetic tree is shown. Statistical support is indicated as posterior probabilities (2,500,000 generations). In all cases, trees were rooted by midpoint rooting and labelled as in Figure 2A. [file 1471-2148-13-96-S3.pdf]

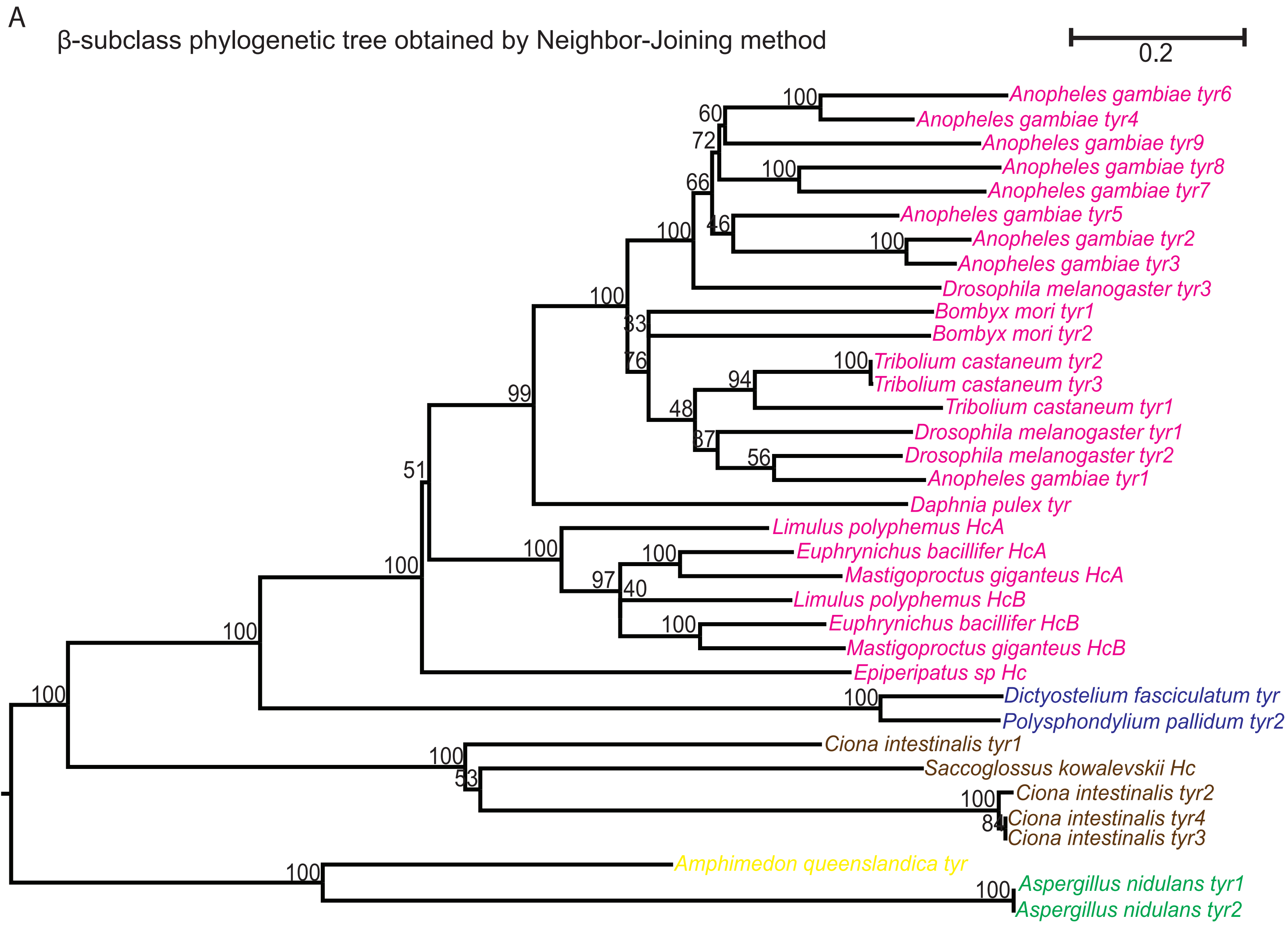

A
β-subclass phylogenetic tree obtained by Neighbor-Joining method
0.2
Anopheles gambiae tyr6
Anopheles gambiae tyr4
Anopheles gambiae tyr9
Anopheles gambiae tyr8
Anopheles gambiae tyr7
Anopheles gambiae tyr5
Anopheles gambiae tyr2
Anopheles gambiae tyr3
Drosophila melanogaster tyr3
Bombyx mori tyr1
Bombyx mori tyr2
Tribolium castaneum tyr2
Tribolium castaneum tyr3
Tribolium castaneum tyr1
Drosophila melanogaster tyr1
Drosophila melanogaster tyr2
Anopheles gambiae tyr1
Daphnia pulex tyr
Limulus polyphemus HcA
Euphrynichus bacillifer HcA
Mastigoproctus giganteus HcA
Limulus polyphemus HcB
Euphrynichus bacillifer HcB
Mastigoproctus giganteus HcB
Epiperipatus sp Hc
Dictyostelium fasciculatum tyr
Polysphondylium pallidum tyr2
Ciona intestinalis tyr1
Saccoglossus kowalevskii Hc
Ciona intestinalis tyr2
Ciona intestinalis tyr4
Ciona intestinalis tyr3
Amphimedon queenslandica tyr
Aspergillus nidulans tyr1
Aspergillus nidulans tyr2

B

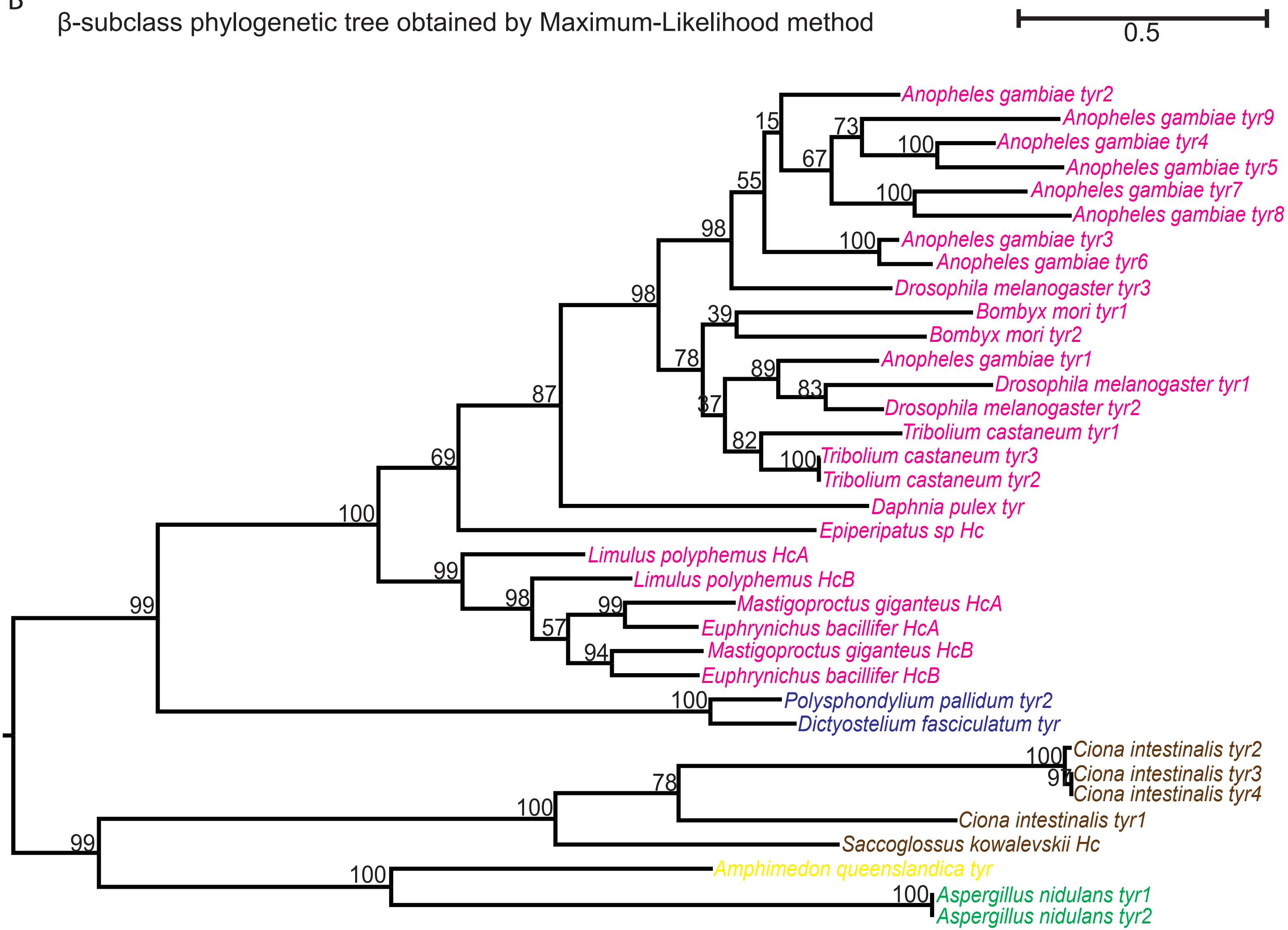
β-subclass phylogenetic tree obtained by Maximum-Likelihood method
0.5
Anopheles gambiae tyr2
Anopheles gambiae tyr9
Anopheles gambiae tyr4
Anopheles gambiae tyr5
Anopheles gambiae tyr7
Anopheles gambiae tyr8
Anopheles gambiae tyr3
Anopheles gambiae tyr6
Drosophila melanogaster tyr3
Bombyx mori tyr1
Bombyx mori tyr2
Anopheles gambiae tyr1
Drosophila melanogaster tyr1
Drosophila melanogaster tyr2
Tribolium castaneum tyr1
Tribolium castaneum tyr3
Tribolium castaneum tyr2
Daphnia pulex tyr
Epiperipatus sp Hc
Limulus polyphemus HcA
Limulus polyphemus HcB
Mastigoproctus giganteus HcA
Euphrynichus bacillifer HcA
Mastigoproctus giganteus HcB
Euphrynichus bacillifer HcB
Polysphondylium pallidum tyr2
Dictyostelium fasciculatum tyr
Ciona intestinalis tyr2
Ciona intestinalis tyr3
Ciona intestinalis tyr4
Ciona intestinalis tyr1
Saccoglossus kowalevskii Hc
Amphimedon queenslandica tyr
Aspergillus nidulans tyr1
Aspergillus nidulans tyr2
15
73
100
67
55
100
98
100
98
39
78
89
83
37
82
100
87
69
100
99
98
99
57
94
99
100
100
91
78
100
99
100
100

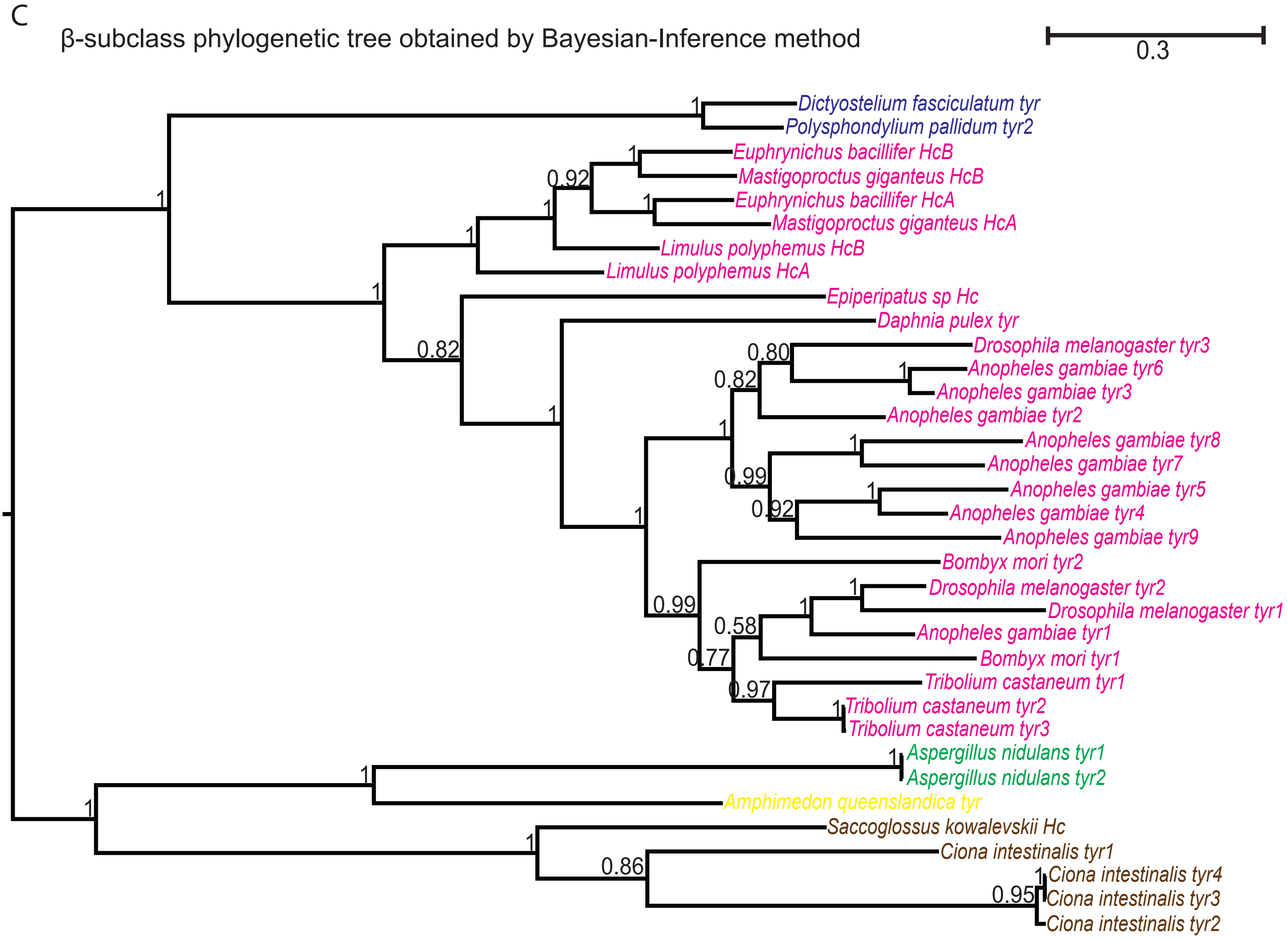

C
β-subclass phylogenetic tree obtained by Bayesian-Inference method
0.3
Dictyostelium fasciculatum tyr
Polysphondylium pallidum tyr2
Euphrynichus bacillifer HcB
Mastigoproctus giganteus HcB
Euphrynichus bacillifer HcA
Mastigoproctus giganteus HcA
Limulus polyphemus HcB
Limulus polyphemus HcA
Epiperipatus sp Hc
Daphnia pulex tyr
Drosophila melanogaster tyr3
Anopheles gambiae tyr6
Anopheles gambiae tyr3
Anopheles gambiae tyr2
Anopheles gambiae tyr8
Anopheles gambiae tyr7
Anopheles gambiae tyr5
Anopheles gambiae tyr4
Anopheles gambiae tyr9
Bombyx mori tyr2
Drosophila melanogaster tyr2
Drosophila melanogaster tyr1
Anopheles gambiae tyr1
Bombyx mori tyr1
Tribolium castaneum tyr1
Tribolium castaneum tyr2
Tribolium castaneum tyr3
Aspergillus nidulans tyr1
Aspergillus nidulans tyr2
Amphimedon queenslandica tyr
Saccoglossus kowalevskii Hc
Ciona intestinalis tyr1
Ciona intestinalis tyr4
Ciona intestinalis tyr3
Ciona intestinalis tyr2
0.92
0.82
0.80
0.82
0.99
0.92
0.99
0.58
0.77
0.97
0.86
0.95
