# Supplementary material for: Origin, evolution and classification of type-3 copper proteins: lineage-specific gene expansions and losses across the Metazoa
Source: BMC Evol Biol. 2013 May 1;13:96. doi: 10.1186/1471-2148-13-96 (PMC3658974; doi:10.1186/1471-2148-13-96)
Supplement: Additional file 4 — Phylogenetic trees: Phylogenetic analyses of the γ-subclass copper proteins. Neighbor-Joining (NJ) phylogenetic tree is shown. Statistical support for each node is indicated as percentage (1,000 bootstrap reanalyses). B. Maximum-Likelihood (ML) phylogenetic tree is shown. Statistical support for each node is indicated as percentage (1,000 bootstrap reanalyses). C. Bayesian Inference (BI) phylogenetic tree is shown. Statistical support is indicated as posterior probabilities (2,500,000 generations). In all cases, trees were rooted by midpoint rooting and labelled as in Figure 2A. [file 1471-2148-13-96-S4.pdf]

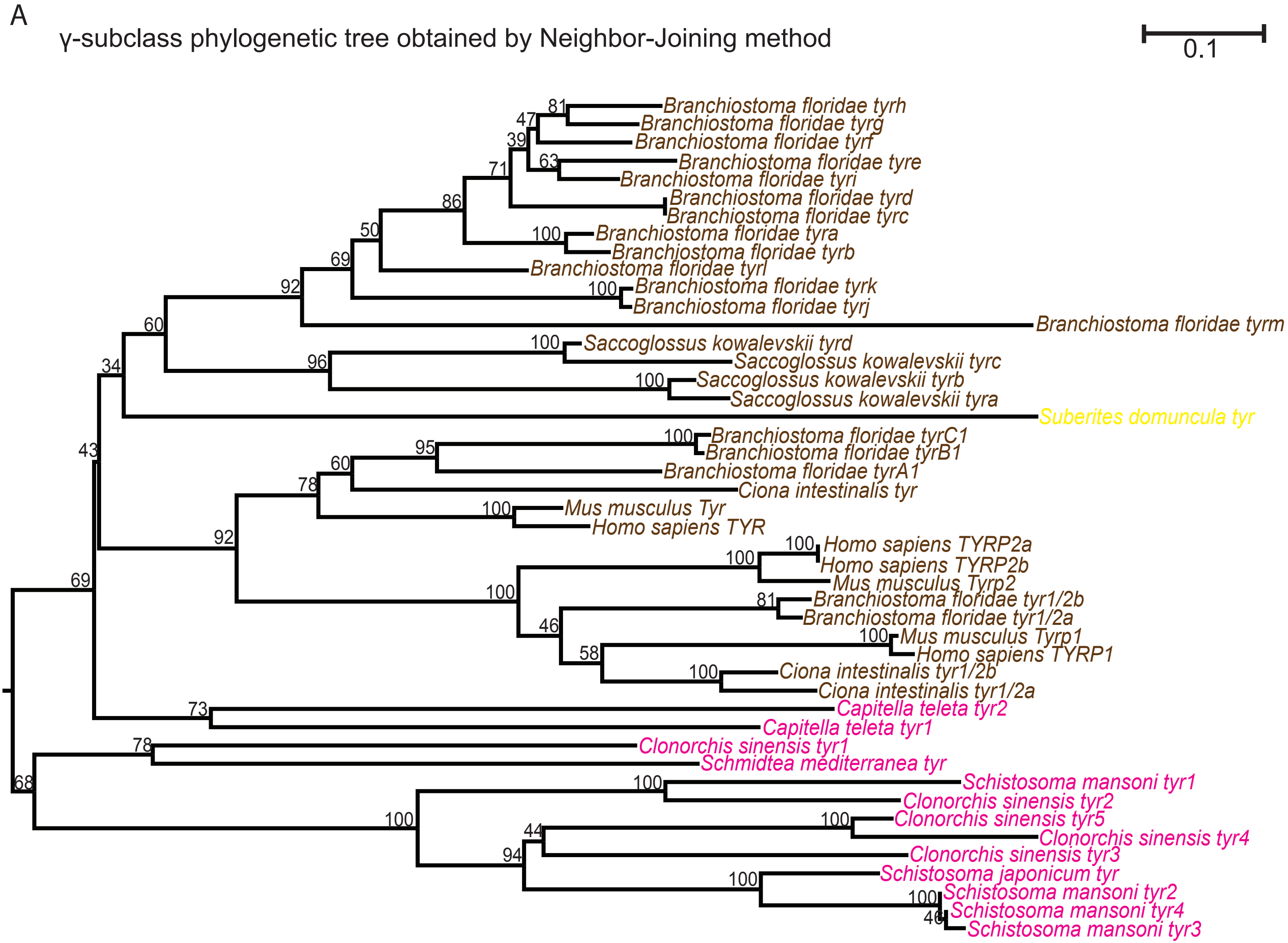
A
γ-subclass phylogenetic tree obtained by Neighbor-Joining method
0.1
Branchiostoma floridae tyrh
Branchiostoma floridae tyrg
Branchiostoma floridae tyrf
Branchiostoma floridae tyre
Branchiostoma floridae tyri
Branchiostoma floridae tyrd
Branchiostoma floridae tyrc
Branchiostoma floridae tyra
Branchiostoma floridae tyrb
Branchiostoma floridae tyrl
Branchiostoma floridae tyrk
Branchiostoma floridae tyrj
Branchiostoma floridae tyrm
Saccoglossus kowalevskii tyrd
Saccoglossus kowalevskii tyrc
Saccoglossus kowalevskii tyrb
Saccoglossus kowalevskii tyra
Suberites domuncula tyr
Branchiostoma floridae tyrC1
Branchiostoma floridae tyrB1
Branchiostoma floridae tyrA1
Ciona intestinalis tyr
Mus musculus Tyr
Homo sapiens TYR
Homo sapiens TYRP2a
Homo sapiens TYRP2b
Mus musculus Tyrp2
Branchiostoma floridae tyr1/2b
Branchiostoma floridae tyr1/2a
Mus musculus Tyrp1
Homo sapiens TYRP1
Ciona intestinalis tyr1/2b
Ciona intestinalis tyr1/2a
Capitella teleta tyr2
Capitella teleta tyr1
Clonorchis sinensis tyr1
Schmidtea mediterranea tyr
Schistosoma mansoni tyr1
Clonorchis sinensis tyr2
Clonorchis sinensis tyr5
Clonorchis sinensis tyr4
Clonorchis sinensis tyr3
Schistosoma japonicum tyr
Schistosoma mansoni tyr2
Schistosoma mansoni tyr4
Schistosoma mansoni tyr3

B

γ-subclass phylogenetic tree obtained by Maximum-Likelihood method

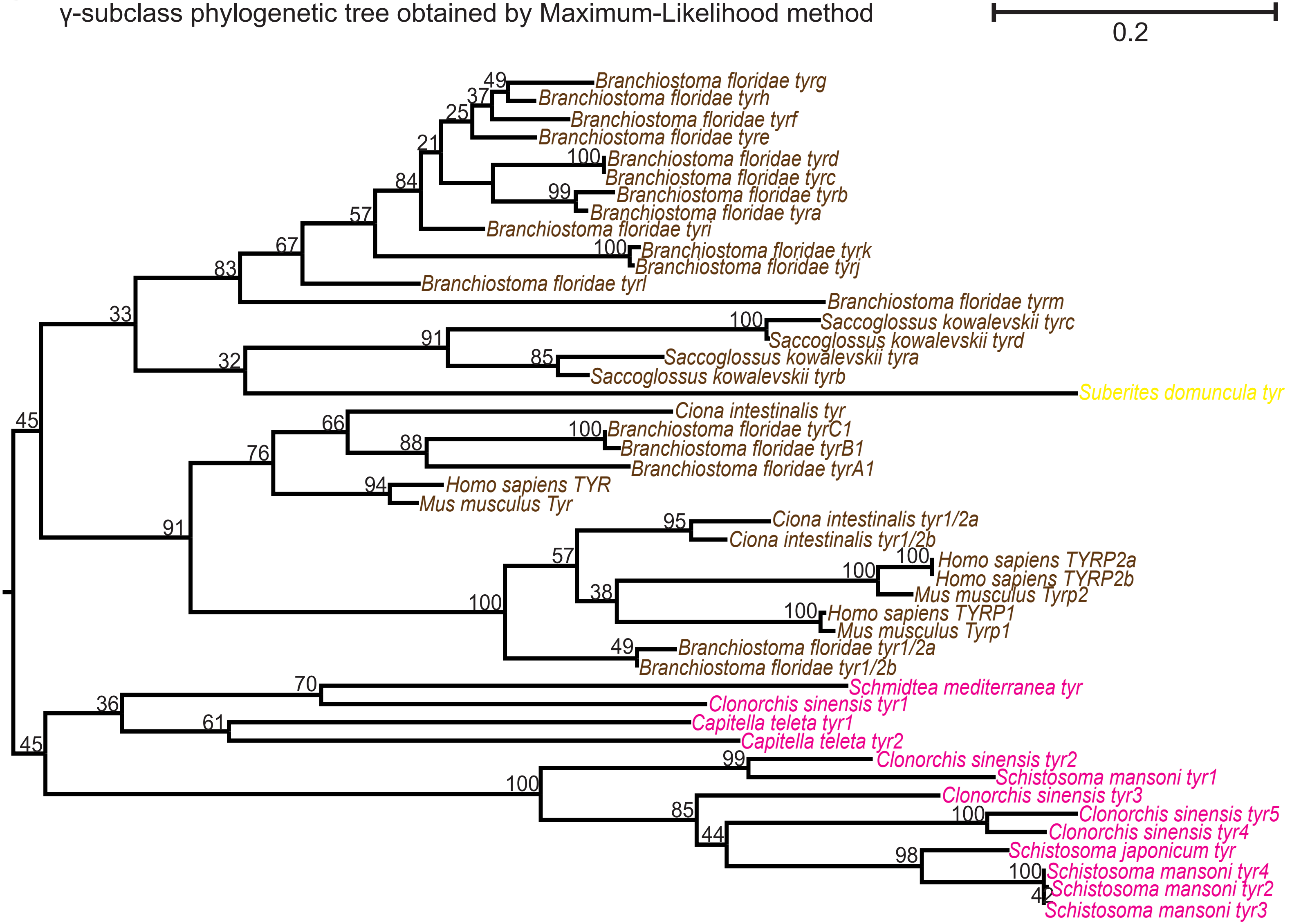

C

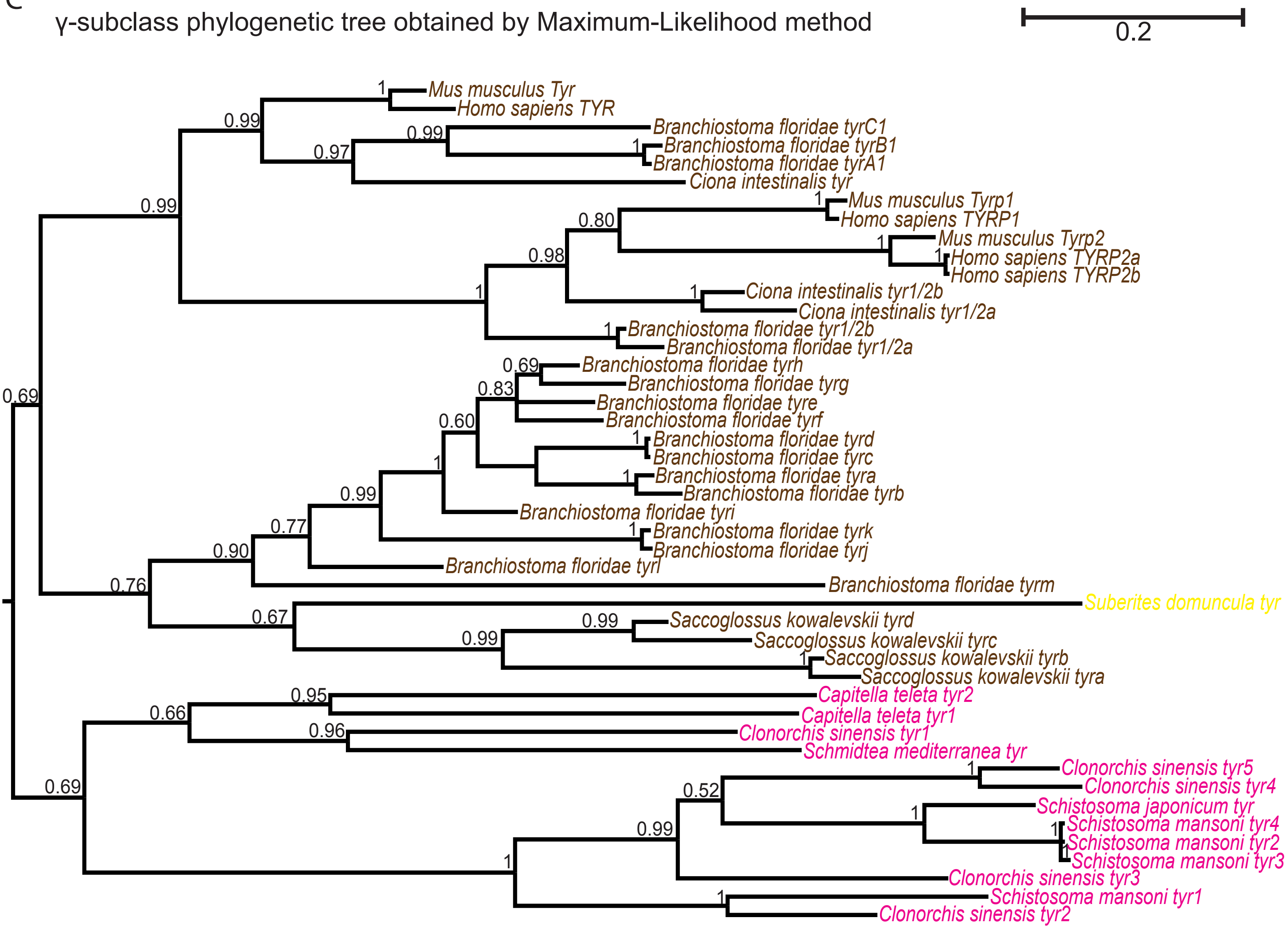
γ-subclass phylogenetic tree obtained by Maximum-Likelihood method
0.2
Mus musculus Tyr
Homo sapiens TYR
Branchiostoma floridae tyrC1
Branchiostoma floridae tyrB1
Branchiostoma floridae tyrA1
Ciona intestinalis tyr
Mus musculus Tyrp1
Homo sapiens TYRP1
Mus musculus Tyrp2
Homo sapiens TYRP2a
Homo sapiens TYRP2b
Ciona intestinalis tyr1/2b
Ciona intestinalis tyr1/2a
Branchiostoma floridae tyr1/2b
Branchiostoma floridae tyr1/2a
Branchiostoma floridae tyrh
Branchiostoma floridae tyrg
Branchiostoma floridae tyre
Branchiostoma floridae tyrf
Branchiostoma floridae tyrd
Branchiostoma floridae tyrc
Branchiostoma floridae tyra
Branchiostoma floridae tyrb
Branchiostoma floridae tyri
Branchiostoma floridae tyrk
Branchiostoma floridae tyrj
Branchiostoma floridae tyrl
Branchiostoma floridae tyrm
Suberites domuncula tyr
Saccoglossus kowalevskii tyrd
Saccoglossus kowalevskii tyrc
Saccoglossus kowalevskii tyrb
Saccoglossus kowalevskii tyra
Capitella teleta tyr2
Capitella teleta tyr1
Clonorchis sinensis tyr1
Schmidtea mediterranea tyr
Clonorchis sinensis tyr5
Clonorchis sinensis tyr4
Schistosoma japonicum tyr
Schistosoma mansoni tyr4
Schistosoma mansoni tyr2
Schistosoma mansoni tyr3
Clonorchis sinensis tyr3
Schistosoma mansoni tyr1
Clonorchis sinensis tyr2
