# Supplementary material for: Origin, evolution and classification of type-3 copper proteins: lineage-specific gene expansions and losses across the Metazoa
Source: BMC Evol Biol. 2013 May 1;13:96. doi: 10.1186/1471-2148-13-96 (PMC3658974; doi:10.1186/1471-2148-13-96)
Supplement: Additional file 5 — Lineage-specific expansion of type-3 copper gene subclasses and their physical linkage: Putative physical linkages of some representatives from α-, β-, and γ-subclasses. Type-3 copper genes that are physically linked. Exons are indicated by boxes; while introns are indicated by lines adjoining these. Copper-binding sites A and B are indicated by black boxes Cu(A) at the left and Cu(B) at the right, respectively. Intergenic distances are indicated in kilobases. All gene structures are drawn to scale but these scales differ between phyla and subclasses. The arrow indicates the direction of transcription for each gene. [file 1471-2148-13-96-S5.pdf]

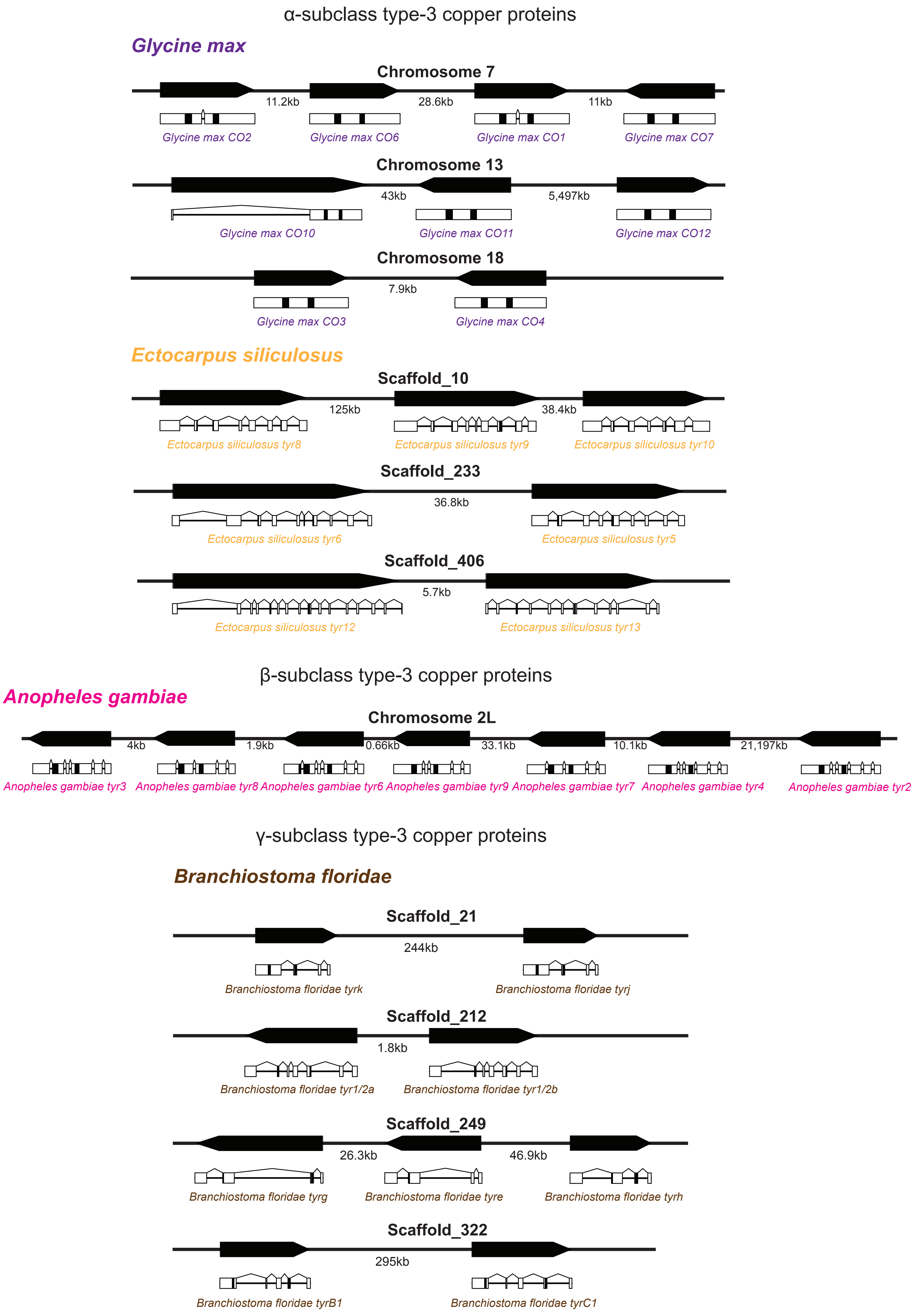
α-subclass type-3 copper proteins
Glycine max
Chromosome 7
11.2kb
28.6kb
11kb
Glycine max CO2
Glycine max CO6
Glycine max CO1
Glycine max CO7
Chromosome 13
43kb
5,497kb
Glycine max CO10
Glycine max CO11
Glycine max CO12
Chromosome 18
7.9kb
Glycine max CO3
Glycine max CO4
Ectocarpus siliculosus
Scaffold_10
125kb
38.4kb
Ectocarpus siliculosus tyr8
Ectocarpus siliculosus tyr9
Ectocarpus siliculosus tyr10
Scaffold_233
36.8kb
Ectocarpus siliculosus tyr6
Ectocarpus siliculosus tyr5
Scaffold_406
5.7kb
Ectocarpus siliculosus tyr12
Ectocarpus siliculosus tyr13
β-subclass type-3 copper proteins
Anopheles gambiae
Chromosome 2L
4kb
1.9kb
0.66kb
33.1kb
10.1kb
21,197kb
Anopheles gambiae tyr3
Anopheles gambiae tyr8
Anopheles gambiae tyr6
Anopheles gambiae tyr9
Anopheles gambiae tyr7
Anopheles gambiae tyr4
Anopheles gambiae tyr2
γ-subclass type-3 copper proteins
Branchiostoma floridae
Scaffold_21
244kb
Branchiostoma floridae tyrk
Branchiostoma floridae tyrj
Scaffold_212
1.8kb
Branchiostoma floridae tyr1/2a
Branchiostoma floridae tyr1/2b
Scaffold_249
26.3kb
46.9kb
Branchiostoma floridae tyrg
Branchiostoma floridae tyre
Branchiostoma floridae tyrh
Scaffold_322
295kb
Branchiostoma floridae tyrB1
Branchiostoma floridae tyrC1
